# Billet Straightening by Three-Point Bending and Its Automation

**DOI:** 10.3390/ma14010090

**Published:** 2020-12-28

**Authors:** Radim Halama, Jan Sikora, Martin Fusek, Jaromír Mec, Jana Bartecká, Renata Wagnerová

**Affiliations:** 1 Department of Applied Mechanics, Faculty of Mechanical Engineering, VŠB—Technical University of Ostrava, 17. listopadu 2172/15, 70800 Ostrava, Czech Republic; jana.bartecka@vsb.cz; 2 Department of Control Systems and Instrumentation, Faculty of Mechanical Engineering, VŠB—Technical University of Ostrava, 17. listopadu 2172/15, 70800 Ostrava, Czech Republic; renata.wagnerova@vsb.cz; 3 ELCOM, a. s., Lomnického 1705/9, 14000 Praha 4-Nusle, Czech Republic; Jaromir.Mec@elcom.cz

**Keywords:** straightening process, three-point bending, FEM, control strategy, billet straightening

## Abstract

This paper presents the current results of cooperation focused on automatic billet straightening machine development. First, an experimental study of three-point bending realized on small specimens is presented to explain the basic ideas of the straightening. Then, the main regimes of straightening and the algorithm itself are described together. Subsequent finite element simulations of operational experiments show the applicability of the developed theory. The significance of material parameters estimation is depicted in this work. At least four parameters have to be properly determined for a new material in the straightening process.

## 1. Introduction

Modern steel factories and enterprises of heavy industry, whose field of activity includes the production of long metallic articles, meet the issue of effective straightening of such products. Typical products that involve straightening during their production technology are billets [1,2], strips [3], railway rails [4], elevator guide rails [5], or more general long linear guideways that enable precise linear motion of machines [6]. For the mentioned commodities, there are only two straightening principles that mostly used in technical practice. The first option is continuous straightening [7,8], where the bar is straightened between two cross-rolling straighteners [9,10] or inside a multi-roller straightening machine [11]. This option, however, is very problematic for straightening bars with large cross-sections [12,13], mainly owing to the requirements of employing mighty bearings.

This article is devoted to the issue of billet straightening, where the second type of straightening is commonly used. The principle of this straightening type relies on three-point bending [14,15], which is more accurate and admits higher dimension variability of straightened billets cross-sections [16]. In ironworks, three-point bending is a necessary operation performed before grinding billets. The straightening of billets is usually done manually by operators in a manual regime based on human vision and joystick control [1].

The straightening process can be automated in accordance with the Industry 4.0 strategy, but this is a challenging task [17,18]. An automatic straightening machine can achieve optimal effectivity only if the straightening algorithm is adopted to the various profile curvatures of the billet (e.g., single-arc shape, “S” shape, or shape with multiple vertices [19]). Each type of billet shape requires a unique approach to the straightening, which minimalizes the time of the process. This is the so-called multi-step straightening mechanism [6,19], for which functionality is necessary to correctly determine the velocity of the straightening force/stroke, the distance of supports, the number of straightening steps, and so on. Different parameter settings return differently straightened billets [5].

The crucial thing is to achieve accurate prediction of spring back [20,21] after releasing the straightening force. To achieve fast calculations, analytical and semianalytical approaches are currently used. The finite element method (FEM) is time-consuming and the solution is dependent on many parameters such as element type, and thus shape functions, geometry, and time discretization (according to the material model implementation), among others. For the purposes of the development of the straightening algorithm, the analytical approach could be inspired by other research works using an analytical solution for spring back prediction. During the last two decades, the strategy of multistep straightening was enhanced for deflected shafts with the circular cross-section by the fuzzy self-learning method [22], for steel wires using genetic programming [23] and for T-section beams using neural networks [24]. The latter approach required finite element simulations to develop the artificial neural network approach. The straightening history should be considered for the prediction of residual stresses, which play an important role in the service of the final products. Ling et al. [25] published an interesting study in this field including the prediction of residual stresses after grinding.

A significant benefit of analytical methods is also the accuracy of the solution, especially when a robust material model is considered in the analysis. Eggertsen and Mattiasson evaluated six cyclic plasticity models for spring back prediction [21]. They showed that the Yoshida–Uemori model [26,27] and its modification can correctly describe the Bauschinger effect, a transient behavior, a permanent softening, and a workhardening stagnation. Hajbarati and Zajkani [28] used the modified Yoshida–Uemori two-surface hardening model [21] to predict the spring back of an advanced high-strength steel. High-strength steels reveal significant spring back. FE analyses of three-point bending experiments were presented, for instance, by Zhao and Lee [29].

The following chapters of this article present the current results in the frame of a long-term project devoted to the development of an automatic billet straightening machine. The machine was designed, constructed, and manufactured by KOMA—Industry s.r.o. for TŘINECKÉ ŽELEZÁRNY a.s. The camera system and visualisation of the straightening was developed by experts from ELCOM, a.s. The focus of the article is to show the basic ideas of the newly proposed algorithm and to explain the necessary optimisation procedure needed to obtain some process parameters. This is very important for achieving reliable and robust straightening.

## 2. Three-Point Bending

**Figure 1:**
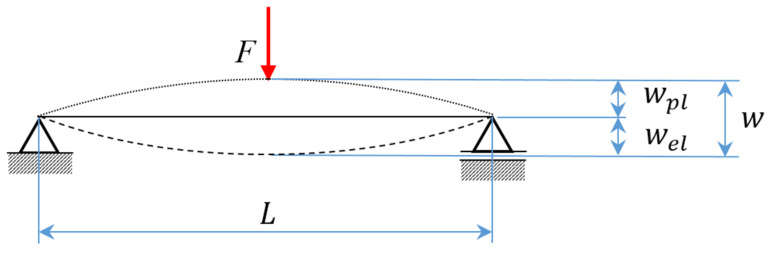
A scheme of a three-point bending case.

First of all, the terminology for three-point bending straightening should be introduced. The simplified situation of the three-point bending case is shown in Figure 1, where the initial shape of the billet is depicted by a dotted line. The maximal deflection w caused by the applied force F can be visualised by the deformed shape of the billet drawn with a dashed line. In the ideal case, the billet shape is straight after spring back, as displayed by the solid line.

In accordance with the additive rule, the total deflection w is composed of plastic deflection $w_{pl}$ and elastic deflection $w_{el}$, thus

(1) $$w=w_{el}+w_{pl}$$

The irreversible deflection $w_{pl}$ is also important input for the algorithm, and it is supposed that it can be accurately measured by a sensory system of an automatic straightening machine for the given distance of supports *L*.

The plastic deflection $w_{pl}$ can be calculated considering an elastic stiffness $k_{el}$ and applied bending force F according to the analogy to Hooke’s law.

(2) $$w_{pl}=w-w_{el}=w-\frac{F}{k_{el}}$$

The elastic stiffness $k_{el}$ is a function of the Young modulus *E*, moment of inertia $I_{z}$, and support distance *L*. We will consider just a square cross-section of the billet in this work, i.e., $I_{z}=D^{4}/12$, where *D* is the dimension of the square cross-section.

A prediction of required total deflection (output quantity of the algorithm) is proposed to be determined from the linear relationship.

(3) $$w=k_{w}w_{pl}+w_{y},$$

where $w_{y}$ and $k_{w}$ are material parameters. Substituting (3) into (2), one can obtain the linear relation between the bending force and total deflection.

(4) $$F=k_{el}\frac{w_{y}}{k_{w}}+k_{el}\left(1-\frac{1}{k_{w}}\right)w=A+B\times w.$$

It can be noted that the parameter $w_{y}$ expresses the total deflection of the billet corresponding to the maximal bending stress in the cross-section for the elastic region of loading, i.e., yield stress $\sigma_{y}$.

## 3. Laboratory Experiments and Their Numerical Simulations

In order to show the idea of the approximation of material response during straightening by three-point bending, an experimental study on three-point bending performed on 51CrV4 material at room temperature will be presented. First, the basic mechanical properties were determined by tensile test; see Table 1. The bending tests were realised on specimens with the square cross-section of variety of dimensions *D* and distances of supports *L*. The proper ratio of *D*/*L* for each bending test had to be determined analytically or numerically.

In this study, finite element method (FEM) was used. The material model introduces the nonlinear kinematic hardening rule of Chaboche [30]. According to Chaboche’s superposition, two back-stress parts are considered to express the back-stress.

(5) $$\alpha =\overset{2}{\underset{i=1}{\sum }}\alpha_{i}=\alpha_{1}+\alpha_{2}$$

and the evolution equation of Armstrong and Frederick [31] for uniaxial loading is

(6) $$d\alpha_{i}=C_{i}d\varepsilon_{p}-\gamma_{i}\alpha_{i}dp$$

where $C_{i}$ and $\gamma_{i}$ are material parameters, $d\varepsilon_{p}$ is the increment of longitudinal plastic strain, and dp is the increment of accumulated plastic strain.

The constitutive equation of the Chaboche model for uniaxial tension is

(7) $$\sigma =\sigma_{y}+\alpha_{1}+\alpha_{2}=\sigma_{y}+\frac{C_{1}}{\gamma_{1}}\left(1-e^{-\gamma_{1}\varepsilon_{p}}\right)+\frac{C_{2}}{\gamma_{2}}\left(1-e^{-\gamma_{2}\varepsilon_{p}}\right)$$

**Figure 2:**
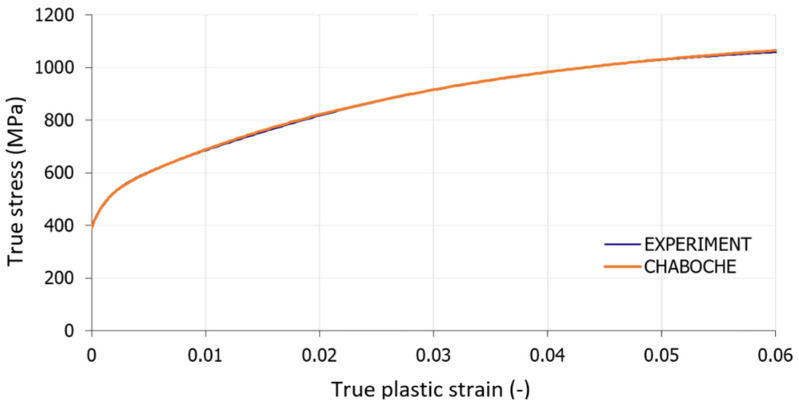
Deformation curve of 51CrV4 material and its approximation by Equation (7).

The tensile curve of the investigated material is used to calibrate the Chaboche model [30] for preliminary simulations by FEM; see Figure 2. All material parameters resulting from a non-linear least-square method application are stated in Table 2. Poisson’s ratio ν = 0.3 was considered in the simulations too.

All FE simulations within this paper were done in ANSYS 2020R1. The goal of the numerical study was to find a proof of the relationship between the total deflection and the plastic deflection described by Equation (3). The square cross-sections of 6 × 6, 8 × 8, 10 × 10, 12 × 12, and 14 × 14 were considered.

**Figure 3:**
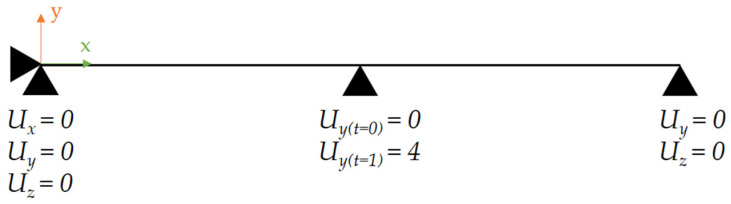
Finite element (FE) model with boundary conditions.

For the discretisation of geometry, the BEAM188 element was used. Boundary conditions applied to the FE model are shown in Figure 3. All nodes of the model are fixed in rotations around the *x*-axis. Ramped displacement with time is applied in the middle of the model in the *y*-direction, leading to maximal displacement of *U_y_* = 4 mm at the end of the computation.

An optimization task was done (parametric study) to get proper distance of the supports for each cross-section dimension *D*. Initially, the distance of supports of 80 mm was chosen for the 6 × 6 specimen. After performing the FE analysis for this case, the dependency of the total deflection on the plastic deflection was evaluated using Equation (2) and approximated by the linear function (3). Then, the largest cross-section of 14 × 14 was considered for simulations by trial and error to gain acceptable correlation with the approximated curve of the first case (total deflection vs. plastic deflection). Other cases, 8 × 8, 10 × 10, and 12 × 12, were solved by repeated FE simulation with an initial guess of the support length supposing the linear relationship between the support distance and cross-section dimension from previous two limit cases.

**Figure 4:**
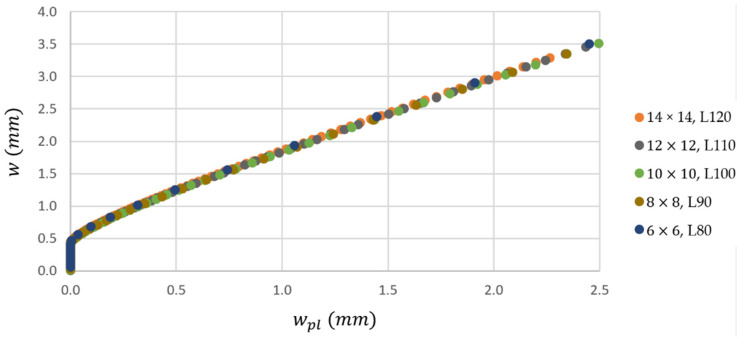
The dependency of total deflection on plastic deflection from the numerical study concerning optimal distances of supports (*L* in the legend) for each cross-section size *D* × *D.*

The resulting curves, which describe the relation between the total deflection and the plastic deflection, are shown in Figure 4. Good overall correlation is achieved for particular cases of cross-sectional dimensions. The dependency is pretty linear in the interval between 0.5 and 2.5 mm of plastic deflection, which confirms the validity of Equation (3). The optimal distances of supports are as follows: 80, 90, 100, 110, and 120 mm (for cross-sectional dimensions of 6 × 6, 8 × 8, 10 × 10, 12 × 12, and 14 × 14).

**Figure 5:**
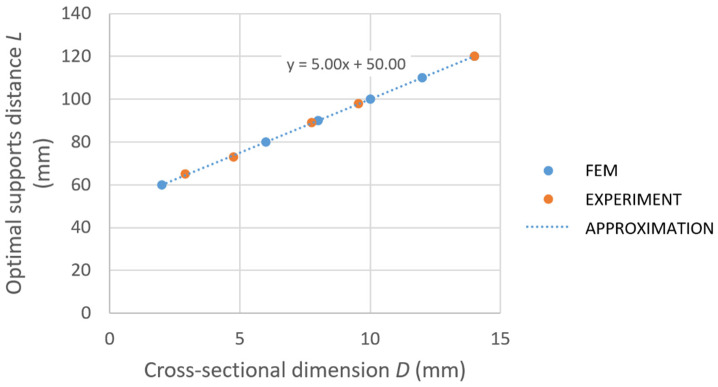
The dependency of the optimal distance of supports *L* on cross-section size *D.* FEM, finite element method.

Based on the numerical study, the curve describing the dependency of the optimal support distance *L* on the cross-sectional dimension *D* of specimens is constructed; see Figure 5. It is clear that the idea of linear dependency of the optimal support distance on the cross-sectional dimension is true. Concerning the available material of billet, the following appropriate dimensions of specimens were selected: 2.9, 4.75, 7.45, 9.55, and 14 mm. The corresponding support distances are as follows: 65, 73, 89, 98, and 120 mm. The specimens for experiments were made by electric discharge machining (EDM) using a portion of the material chosen from the same position of the billet cross-section as for tensile tests.

**Figure 6:**
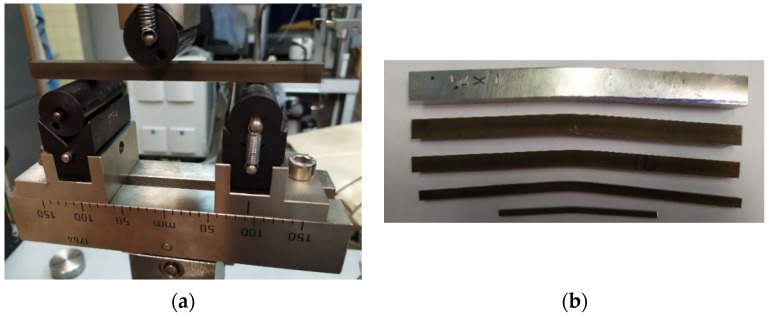
Photos from the three-point bending test: the whole setup (**a**) and selected deformed specimens (**b**).

All experiments were realized using a TESTOMETRIC M500-50CT universal testing machine. The position rate was 5 mm per minute. Deflection was measured as the position of the crossbar. A photo from a three-point bending test realization is shown in Figure 6, where the deformed shapes of specimens are also presented.

**Figure 7:**
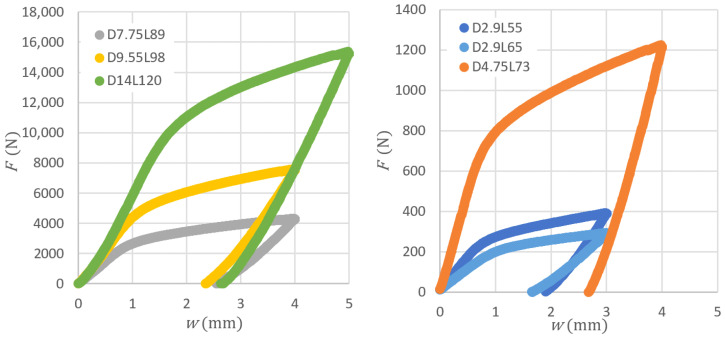
Force response to total deflection for all considered cases.

Obtained bending force versus total deflection diagrams are shown in Figure 7. The target total deflection (position of crossbar) was 3 mm for *D* = 2.9, 5 mm for *D* = 14, and 4 mm for all others.

**Figure 8:**
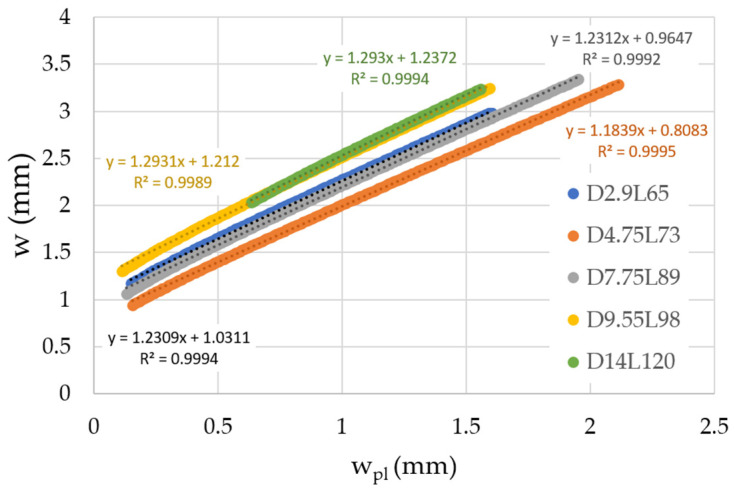
Dependences of total deflection on plastic deflection evaluated from three-point bending tests.

For eventual straightening of billets with different cross-sectional dimensions, it is important to investigate how the dependences of the total deflection on the plastic deflection differ for individual cross-sections, as presented in Figure 8.

An important finding from the performed experimental study is the fact that the slope $k_{w}$ remains approximately the same even though the cross-sections are significantly different in their dimensions. The curves on the graph shown in Figure 8 differ only in the vertical offset. It should be noted that a slight nonlinearity is present in the initial part of the curve of total deflection versus plastic deflection. However, the straightening of billets will be done only in positions where it makes sense. The interventions will be proposed only for significant deviation from a straight line created between supports based on billet shape captured by the camera system.

## 4. Camera System

**Figure 9:**
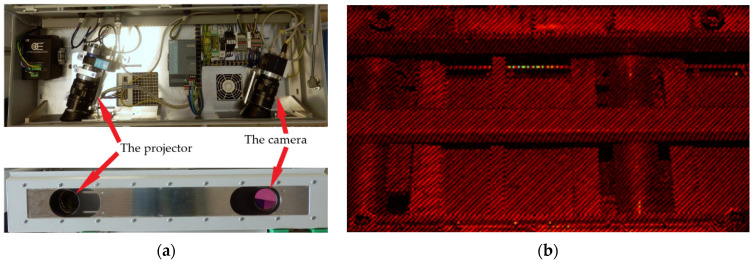
The case for a camera-projector subsystem (**a**), and scanned sector of the straightening machine with the billet (**b**).

As mentioned above, accurate measurement of the initial billet shape is important to achieve reliable results in the straightening process. At the beginning of the straightening process, a profile of the billet is scanned by the camera system for a given side of the billet. The sensory system is composed of eight 2D monochrome cameras. Each camera is paired with a projector that projects a strip pattern on the scanned billet (Figure 9). The projectors are involved into the scanning process to eliminate poor contrast between the billet and the straightening machine and improve the overall quality of received data that directly influence the quality of the curve representing the billet shape.

**Figure 10:**
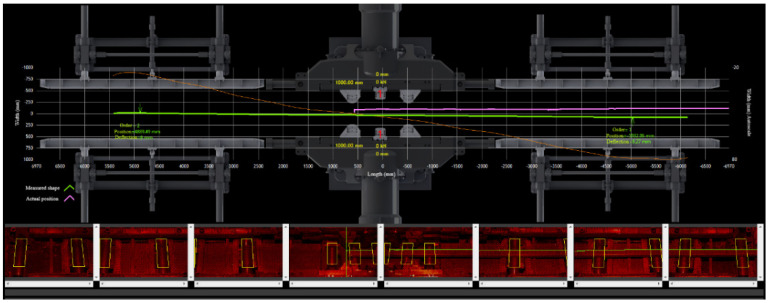
Visualization of initial billet shape and proposed strokes in the technology.

The cameras are equally distributed above the straightening machine to capture the whole area of the press technology (14 m × 1 m). Each camera captures a sector of technology with a length of 2.2 m. Pictures from neighbour cameras are overlapping, so we can get a picture of the whole billet by continuous junction of pictures from individual sections. By processing the picture of the whole billet, we can detect one of the upper edges of the billet. This is crucial for obtaining the curve representing the profile of the billet. An example of a screen visible for operators with subsequently proposed two strokes is shown in the Figure 10.

## 5. Straightening Algorithm

The straightness of the billet is defined by two criteria that determine the type of the billet based on its shape. Both parameters direct the straightening regime subsequently applied in the algorithm.

**Figure 11:**
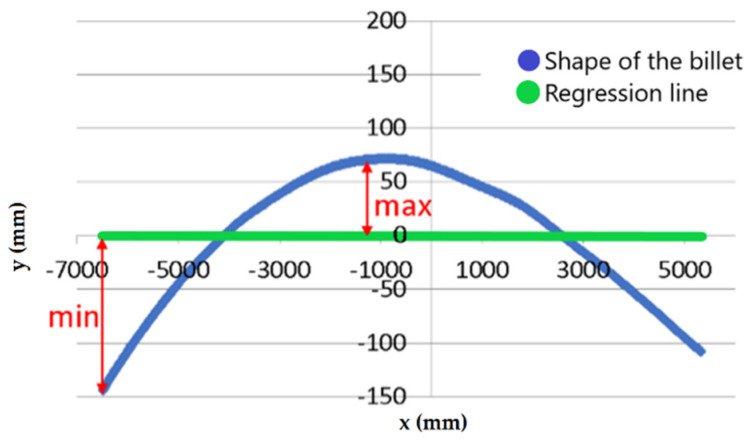
Scheme of gathering parameter *p*_1_.

The first parameter is the sum of the maximum and minimum deviation from the linear regression line (Figure 11) considering the whole curve of the billet. It is marked as *p*_1_ in the algorithm. The critical value of parameter *p*_1_ is marked as *p*_1*crit*_ and should be appropriately chosen according to the current billet length.

The second parameter called *p*_2_ contains the value of maximum deviation on a 1 m segment. This is obtained when the 1 m segment is virtually moved along the whole length of the curve. The deviation on the 1 m segment is determined by the maximum deviation of the curve point from the line connecting the two ending points of the segment. The critical value of the parameter *p*_2_ will be marked as *p*_2*crit*_ and influences the output accuracy of the straightening process.

The objective of the straightening algorithm is to straighten the billet i.e., to reduce both billet parameters below their critical values. The definition of a straight billet depends on subsequent technological processes and customer requirements. The most commonly applied technological process is grinding. Currently acceptable values by customers are *p*_1*crit*_ = 15 mm (12 m billet length) and *p*_2*crit*_ = 2 mm.

**Figure 12:**
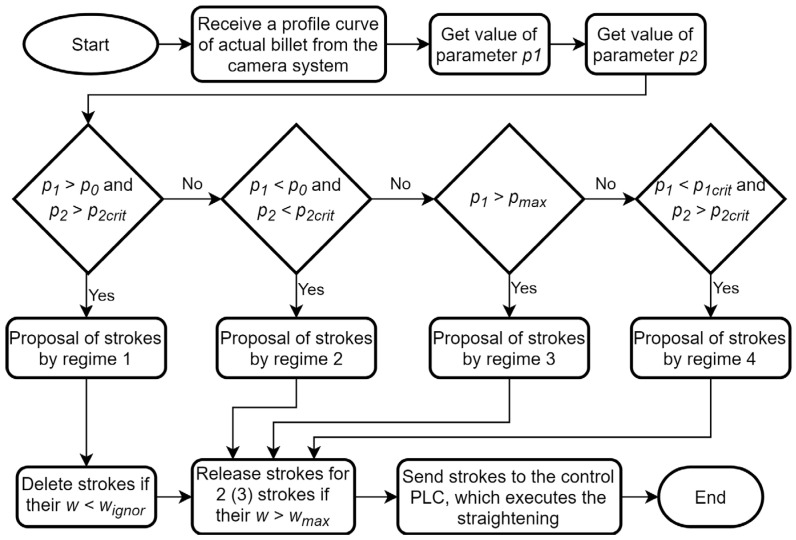
Flowchart of the straightening algorithm part proposing strokes (PLC—Programmable Logic Controller).

Four different straightening regimes of the algorithm are currently applied. The regime of the straightening algorithm is chosen based on the parameters mentioned above, supplemented by *p*_0_ and *p_max_*, which help to distinguish slightly curved billets and strongly crooked ones, respectively. The values of *p*_0_ and *p_max_* are constant for a given material. The regime of the algorithm is chosen based on the billet shape according to schema of the algorithm; see Figure 12.

The first regime is applied in the case of valid conditions *p*_1_ > *p*_0_ and *p*_2_ > *p*_2*crit*_. This variant is usually the most effective one for “snake-like” billets. The billet is divided into particular sections with a length of 1 m. In each section, a regression line is determined and the value of w is calculated by Equation (3) based on the value of $w_{pl}$, which is given from the measured shape within the 1 m segment. If w > $w_{ignor}$ then an intervention is performed in the given position. This variant of straightening is usually quite time-consuming for a large number of interventions, thus the value of $w_{ignor}$ should be optimized to achieve an acceptable speed of straightening without compromising accuracy. The parameter $w_{ignor}$ has the meaning of the minimal applied stroke in the first regime.

The second regime of the algorithm is chosen for *p*_1_ < *p*_0_ and *p*_2_ < *p*_2*crit*_. The billet can be categorized as slightly curved “S-shaped” billet or “single-arc” type billet. Therefore, it is straightened either by two strokes or just one.

The third regime of straightening is used in the interval *p*_1_ > *p_max_*. The condition corresponds to a strongly crooked billet. The straightening is boosted according to the given material. An empirically determined multiplier is used for all strokes calculated by Equation (3).

The last regime is when *p*_1_ < *p*_1*crit*_ and *p*_2_ > *p*_2*crit*_. It is evident from the condition that it usually corresponds to the case where the billet is curved in just one place. The largest deviation on the 1 m segment is found and the stroke is proposed using Equation (3).

The detailed flowchart of the complete straightening algorithm is shown in Figure 12. The part of the algorithm determining the positions and stroke proposals was written in NI LabView 2014 interface.

## 6. Operational Experiments and Their Numerical Simulations

To show the efficiency of the straightening algorithm in the second regime of the algorithm, two exemplar billets made from 100Cr6 material with a cross-section of 150 mm × 150 mm corresponding to “single-arc” and “S-shaped” type were selected for reporting as operational experiments.

**Figure 13:**
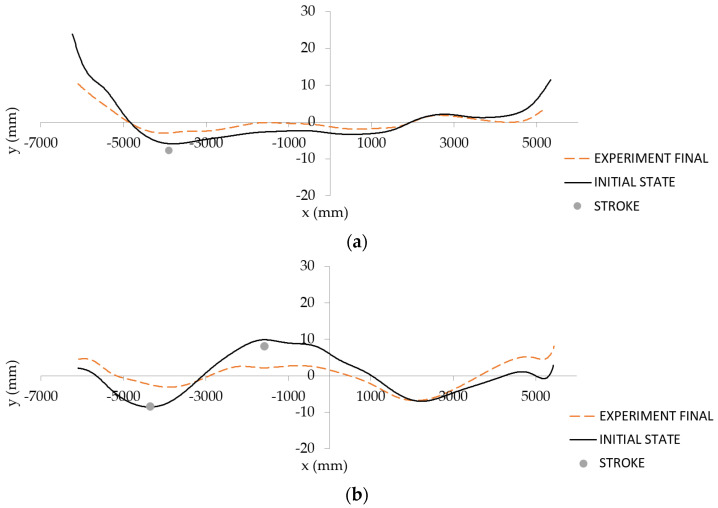
Initial and final shapes of two exemplary billets from operational experiments: “single-arc” type (**a**) and “S-shaped” type (**b**).

Input parameters of the first billet shape were *p*_1_ = 29.8 mm and *p*_2_ = 2.4 mm. After one stroke application (w=7.76 mm, in the position x=−3889 mm), the output parameters evaluated by the sensory system were 11.9 and 1.3 mm. The initial and final shape of the first exemplar billet is shown in Figure 13a. The input parameters of the second billet shape were *p*_1_ = 20.8 mm and *p*_2_ = 2.4 mm. After two strokes application (w=8.37 mm, in the position x=−4350 mm and w=−8.1 mm, in the position x=−1580 mm), the output parameters evaluated by the sensory system were 14.4 and 1.8 mm. The initial and final shape of the second exemplar billet is shown in Figure 13b.

**Figure 14:**
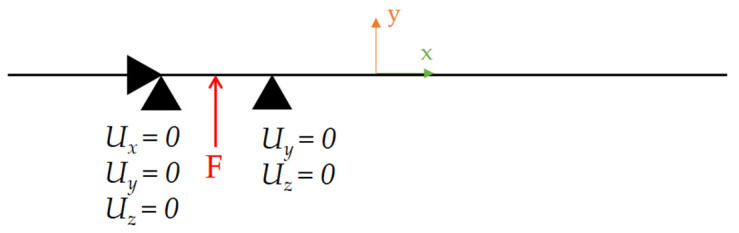
Boundary conditions for the first step of “S-shaped” billet simulation.

Finite element simulations were performed using the same strategy as in Section 3. Each billet was modelled using a spline curve created from points with an increment of 10 mm in the *x*-axis based on data obtained from the camera system. All nodes of the FE model are fixed in rotations around the *x*-axis. In the simulation of the “S-shaped” billet, two load steps were used. First, the boundary conditions of load step one will be described. Displacement boundary conditions were applied according to Figure 14. The force applied in the middle of the support distance (*L* = 1 m) was applied as a linear function of time. The maximal size of force is reached for 1 s with the corresponding value calculated from Equation (4). Then, a linear decrease of force to 10 N (because of convergency) is applied during the unloading phase, which ends after 2 s. In the second load step of the simulation, the maximal force is applied for 3 s considering Equation (4), and displacements were fixed similarly as shown in Figure 14 (supports moved to the new positions). The unloading phase is finished at 4 s with 10 N of force in the computation. The boundary conditions for the “single-arc” billet straightening simulation were analogous to those described for load step one of the “S-shaped” billet straightening simulation.

**Figure 15:**
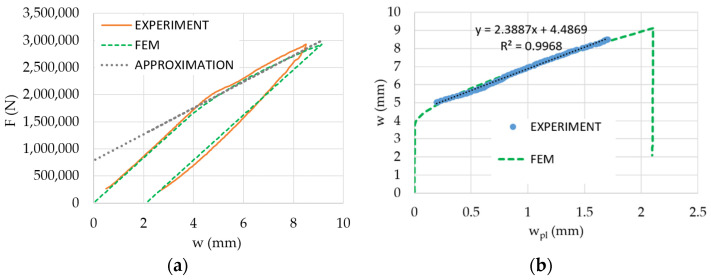
Prediction by the Chaboche model: force vs. total deflection including approximation by Equation (4) (**a**) and total deflection vs. plastic deflection (**b**).

The Chaboche material model was calibrated to give an acceptable response of force for a given total deflection and to give a similar curve of total deflection versus plastic deflection; see Figure 15. Poisson’s ratio ν = 0.3 was considered in both simulations. All other material parameters of the Chaboche model are stated in Table 3.

**Figure 16:**
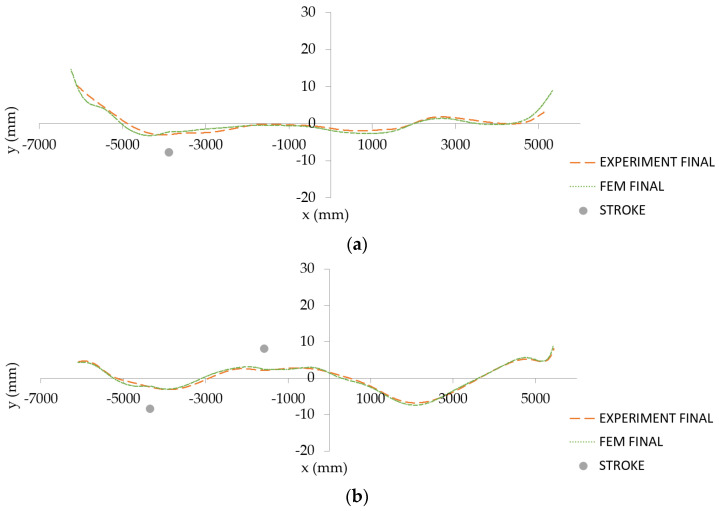
Comparison of experimental and predicted final shapes of two exemplary billets: “single-arc” type (**a**) and “S-shaped” type (**b**).

A comparison of experimental and predicted final billet shapes is provided in Figure 16. It is clearly shown that the strategy for numerical prediction gives acceptable results.

**Figure 17:**
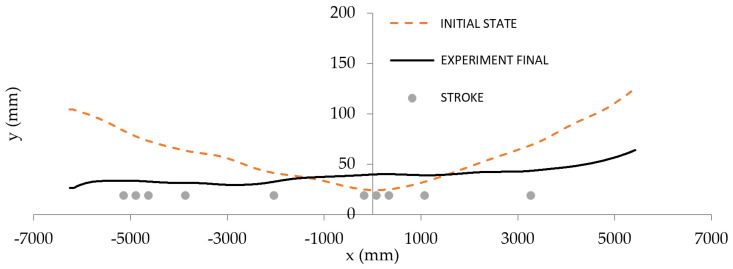
Shape of the third exemplary billet before straightening (solid curve) and after straightening (dashed curve) in regime 3.

An exemplary result of regime 3 application on a very curved billet is shown in Figure 17. The input parameters of the third considered billet were *p*_1_ = 95.4 mm and *p*_2_ = 2.7 mm. After straightening, the output parameters evaluated by the sensory system were 17.8 mm and 1.8 mm. Thus, the straightening in regime 2 followed.

**Figure 18:**
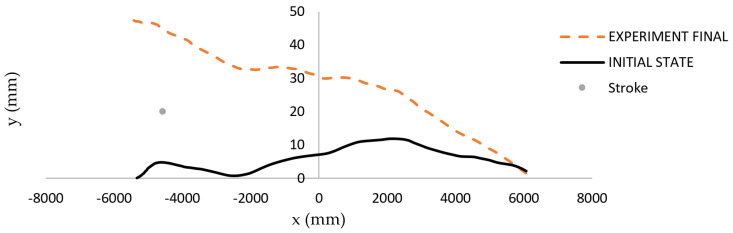
Shape of the fourth exemplary billet before straightening (solid curve) and after straightening (dashed curve) in regime 4.

The results of the fourth regime, which treats the situation of significant curvature in one place, will be presented on the exemplary billet with input parameters *p*_1_ = 10.3 mm and *p*_2_ = 2.4 mm. The stroke of w=7.9 mm was realized in the position x=−4575 mm. The output values observed after straightening were *p*_1_ = 10.5 mm and *p*_2_ = 1 mm. The initial and final shapes are displayed together with the symbol of applied stroke in Figure 18.

The efficiency of the first regime in the straightening algorithm will be shown on the exemplar “snake-like” billet with input parameters *p*_1_ = 66.2 mm and *p*_2_ = 3.9 mm. After the first application of regime 1 (strokes w=9 mm for x=−5731 mm, w=11 mm for x=−5211 mm, w=10.3 mm for x=−3951 mm, w=9.3 mm for x=−2981 mm, w=8.9 mm for x=−1801 mm, w=9 mm for x=−401 mm, and w=8.4 mm for x=839 mm), the output parameters evaluated by the sensory system were 45.8 mm and 3 mm, which means that the first regime was applied again. The second application of regime 1 (strokes w=9 mm for x=−5731 mm, w=11 mm for x=−5211 mm, w=10.3 mm for x=−3951 mm, w=9.3 mm for x=−2981 mm, w=8.9 mm for x=−1801 mm, w=9 mm for x=−401 mm, and w=8.4 mm for x=839 mm) gave acceptable output parameters of *p*_1_ = 8.6 mm and *p*_2_ =1 mm.

**Figure 19:**
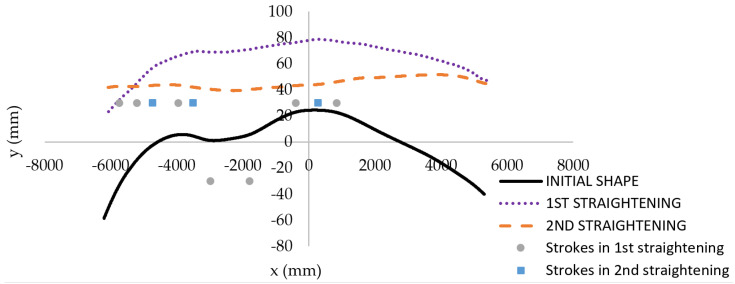
Shape of the “snake-like” exemplary billet before straightening (solid curve), after first straightening (dotted curve), and after second straightening (dashed curve) in regime 1.

**Figure 20:**
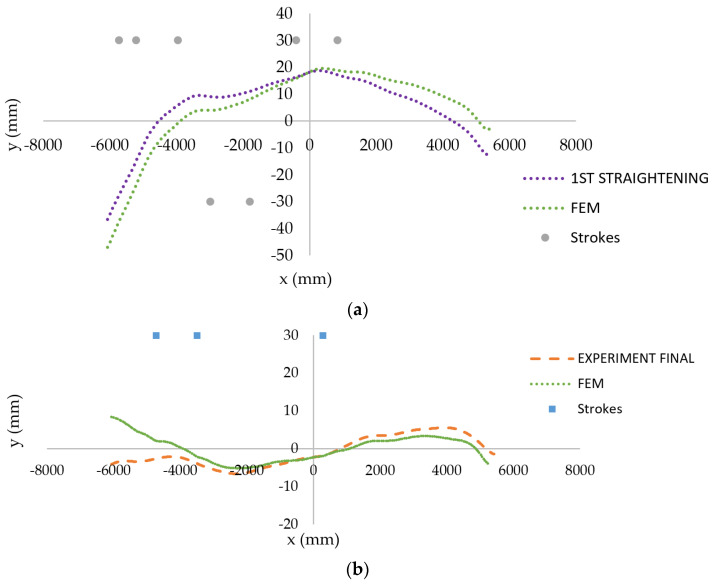
Comparison of experimental and predicted shapes of the exemplary “snake-like” billet: after first straightening (**a**) and after second straightening (**b**).

The initial and straightened experimental shapes of the “snake-like” exemplar billet are shown in Figure 19. The results of corresponding FE simulations are presented in Figure 20. Strokes proposed by the algorithm in reality were applied in particular load-steps in a stroke by stroke manner. The boundary conditions used in each load-step of simulations were analogous to those presented in Figure 14.

## 7. Conclusions

An automatic billet straightening machine was developed in cooperation between the university and industrial companies. The nature of the algorithm proposing interventions during the straightening process is described in this scientific work.

While the algorithm currently works for a 150 × 150 mm^2^ cross-section, it can be expanded into a more general form based on findings shown in the laboratory experiments (Section 3) to be applicable for the straightening of billets with various cross-section sizes. In that case, the most important outcome of the laboratory study is the possibility of constant value consideration for the plastic hardening parameter $k_{w}$. Then, it is necessary to increase the support distance for a larger cross-section according to the linear approximation shown in the Figure 5. The material parameter $w_{y}$ depends on the yield strength of the material, Young modulus *E*, and the cross-section dimension of the billet. Both material parameters, $k_{w}$ and $w_{y}$, must be properly identified for the considered material of billet from the force versus total deflection curve obtained for the chosen cross-section size. The algorithm itself is based on the assumption of a linear relationship between the total deflection and the plastic deflection. In fact, there is a slight nonlinearity for very small values of plastic deflection and this interval corresponds to the nonlinear part of the force versus total deflection diagram (for example in Figure 7). However, this interval is rarely used in the straightening algorithm. There is the parameter $w_{ignor}$, which corresponds to the minimal applied stroke for regime 1. In other regimes, it was experimentally proven that even a small intervention can help to straighten the billet (“single-arc” or “S-shaped” billets, usually).

Numerical simulations of operational experiments were done based on the Chaboche material model with two backstress parts to show the relevance of the algorithm. The basic regimes of the straightening algorithm considering different shapes of the billet were described. The algorithm was adopted on chosen steels in The New Long Billet Treatment Plant of Třinecké železárny a.s. The process and material parameters are optimised using a Python code. The billet straightening strategy currently works properly for ten materials under consideration.

The next step of research is the application of rigid body movement calculations (a simplified approach to predict the impact of performed stroke on the billet shape change) to speed up the straightening process by minimizing the necessity of scanning.

## Figures and Tables

**Table 1:** Mechanical properties of 51CrV4 material obtained from the tensile tests.

Quantity	Averaged Values
Yield strength *R_p_*_0,2_ (MPa)	523
Ultimate strength *R_m_* (MPa)	1005
Young modulus *E* (MPa)	207,000
Ductility (%)	15.6

**Table 2:** Material parameters of the Chaboche model for 51CrV4 material.

E	$\sigma_{y}\;(\mathrm{MPa})$	$C_{1}\;(\mathrm{MPa})$	$\gamma_{1}\;(-)$	$C_{2}\;(\mathrm{MPa})$	$\gamma_{2}\;(-)$
207,000	391	97,000	877	22,000	34

**Table 3:** Material parameters of the Chaboche model for 100Cr6 material.

E	$\sigma_{y}\;(\mathrm{MPa})$	$C_{1}\;(\mathrm{MPa})$	$\gamma_{1}\;(-)$	$C_{2}\;(\mathrm{MPa})$	$\gamma_{2}\;(-)$
220,000	550	202,000	802	61,600	44

## References

[1] Fusek M., Halama R., Poruba Z. Calibration of material parameters during billet straightening. Proceedings of the EAN 2017-55th Conference on Experimental Stress Analysis 2017.

[2] Halama R., Sikora J., Fusek M., Marek M., Bartecká J., Guráš R., Wagnerová R., Mahdal M. Algorithms for Automatic Billet Straightening Machine. Proceedings of the EAN 2020—58th Conference on Experimental Stress Analysis 2020.

[3] Yi G., Wang Z., Hu Z. (2020). A novel modeling method in metal strip leveling based on a roll-strip unit. Math. Probl. Eng..

[4] Kaiser R., Stefenelli M., Hatzenbichler T., Antretter T., Hofmann M., Keckes J., Buchmayr B. (2015). Experimental characterization and modelling of triaxial residual stresses in straightened railway rails. J. Strain Anal. Eng. Des..

[5] Wang K., Wang B., Yang C. (2011). Research on the Multi-Step Straightening for the Elevator Guide Rail. Procedia Eng..

[6] Zhang Y., Lu H., Zhang X., Ling H., Fan W., Wei Q., Lian Y. (2019). A novel control strategy for the multi-step straightening process of long/extra-long linear guideways. Proc. Inst. Mech. Eng. Part. C J. Mech. Eng. Sci..

[7] Petruška J., Návrat T., Šebek F. (2016). Novel approach to computational simulation of cross roll straightening of bars. J. Mater. Process. Technol..

[8] Wu B.J., Chan L.C., Lee T.C., Ao L.W. (2000). A study on the precision modeling of the bars produced in two cross-roll straightening. J. Mater. Process. Technol..

[9] Fan Q.-H., Ma Z.-Y., Ma L.-D., Lei J.-Y. (2020). Study on roller shape of two-roll straightening of titanium alloy based on rolling and reverse bending theory. Suxing Gongcheng Xuebao J. Plast. Eng..

[10] Ma L., Du Y., Liu Z., Ma L. (2019). Design of continuous variable curvature roll shape and straightening process research for two-roll straightener of bar. Int. J. Adv. Manuf. Technol..

[11] Petruška J., Návrat T., Šebek F., Benešovský M. Optimal intermeshing of multi roller cross roll straightening machine. Proceedings of the 19th International ESAFORM Conference on Material Forming.

[12] Zhang Z.-Q. (2016). Prediction of Maximum Section Flattening of Thin-walled Circular Steel Tube in Continuous Rotary Straightening Process. J. Iron Steel Res. Int..

[13] Zhang Z.Q., Yan Y.H., Yang H.L. (2016). A simplified model of maximum cross-section flattening in continuous rotary straightening process of thin-walled circular steel tubes. J. Mater. Process. Technol..

[14] De Morais A.B. (2020). A thick bondline beam model for the adhesively bonded 3-point bending specimen. Int. J. Adhes. Adhes..

[15] Lu H., Zang Y., Zhang X., Zhang Y., Li L. (2020). A General Stroke-Based Model for the Straightening Process of D-Type Shaft. Processes.

[16] Essa A.-E., Nasr M., Ahmed M. Variation of The Residual Stresses and Springback in Sheet Bending from Plane-Strain to Plane-Stress Condition using Finite Element Modelling. Proceedings of the International Conference on Applied Mechanics and Mechanical Engineering, Military Technical College Kobry El-Kobbah.

[17] Xiao-Lin W., Zhao-Bo Q. Analyzing an Implemented Mechanism of Intelligent System and Its Work Flow for Straightening Machine of Heavy Beam. Proceedings of the 2010 International Conference on Intelligent System Design and Engineering Application.

[18] Zhang Y., Lu H., Ling H., Lian Y., Ma M. (2018). Analytical Model of a Multi-Step Straightening Process for Linear Guideways Considering Neutral Axis Deviation. Symmetry.

[19] Zhang Y., Lu H., Wang Y., Zhang X., Zhang J., Ling H. (2019). Variable Span Multistep Straightening Process for Long/Extra-Long Linear Guideways. IEEE Access.

[20] Jin L., Yang Y.-F., Li R.-Z., Cui Y.-W., Jamil M., Li L. (2020). Study on Springback Straightening after Bending of the U-Section of TC4 Material under High-Temperature Conditions. Materials.

[21] Eggertsen P.-A., Mattiasson K. (2011). On the identification of kinematic hardening material parameters for accurate springback predictions. Int. J. Mater. Form..

[22] Kim S.C., Chung S.C. (2002). Synthesis of the multi-step straightness control system for shaft straightening processes. Mechatronics.

[23] Balic J., Nastran M. (2002). An on-line predictive system for steel wire straightening using genetic programming. Eng. Appl. Artif. Intell..

[24] Song Y., Yu Z. (2013). Springback prediction in T-section beam bending process using neural networks and finite element method. Arch. Civ. Mech. Eng..

[25] Ling H., Yang C., Feng S., Lu H. (2020). Predictive model of grinding residual stress for linear guideway considering straightening history. Int. J. Mech. Sci..

[26] Yoshida F., Uemori T. (2003). A model of large-strain cyclic plasticity and its application to springback simulation. Int. J. Mech. Sci..

[27] Yoshida F., Uemori T., Fujiwara K. (2002). Elastic-plastic behaviour of steel sheets under in-plane cyclic tension-compression at large strain. Int. J. Plast..

[28] Hajbarati H., Zajkani A. (2019). A novel analytical model to predict springback of DP780 steel based on modified Yoshida-Uemori two-surface hardening model. Int. J. Mater. Form..

[29] Zhao K.M., Lee J.K. (2002). Finite element analysis of the threepoint bending of sheet metals. J. Mater. Process. Technol..

[30] Chaboche J.L., Lemaitre J. (1990). Mechanics of Solid Materials.

[31] Armstrong P.J., Frederick C.O. (1996). A Mathematical Representation of the Multiaxial Bauschinger Effect, G.E.G.B. Report RD/B/N.

